# Are concomitant treatments confounding factors in randomized controlled trials on intensive blood-glucose control in type 2 diabetes? a systematic review

**DOI:** 10.1186/1471-2288-13-107

**Published:** 2013-08-30

**Authors:** Rémy Boussageon, Irène Supper, Sylvie Erpeldinger, Michel Cucherat, Theodora Bejan-Angoulvant, Behrouz Kassai, Catherine Cornu, François Gueyffier

**Affiliations:** 1 Département de Médecine Générale, Université Claude Bernard Lyon 1, Lyon F-69008, France; 2 UMR 5558, Laboratoire de Biométrie et Biologie Evolutive, CNRS, Université Claude Bernard Lyon 1, Villeurbanne F-69100, France; 3 Service de Pharmacologie Clinique, Centre Hospitalier Régional et Universitaire de Tours, Tours, France; 4 UMR 7292, CNRS, Université François Rabelais, Tours, France; 5 Clinical Investigation Centre, INSERM CIC201, Louis Pradel Hospital, Bron 69500, France; 6 Hospices Civils de Lyon, Service de Pharmacologie Clinique, Hôpital Louis Pradel, Bron 69500, France

**Keywords:** Blood glucose, Concomitant treatment, Observer bias, Randomized controlled trial, Type 2 diabetes mellitus

## Abstract

**Background:**

Open-label, randomized controlled trials (RCTs) are subject to observer bias. If patient management is conducted without blinding, a difference between groups may be explained by other factors than study treatment. One factor may come from taking concomitant treatments with an efficacy on the studied outcomes. In type 2 diabetes, some antihypertensive or lipid-lowering drugs are effective against diabetic complications. We wanted to determine if these concomitant treatments were correctly reported in articles of RCTs on type 2 diabetes and if they might have influenced the outcome.

**Methods:**

We performed a systematic review using Medline, Embase, and the Cochrane Library (from January 1950 to July 2010). Open-label RCTs assessing the effectiveness of intensive blood-glucose control in type 2 diabetes were included. We chose five therapeutic classes with proven efficacy against diabetes complications: angiotensin-converting enzyme inhibitors (ACEIs), angiotensin II receptor antagonists (AIIRAs), fibrates, statins, and aspirin. Differences between concomitant treatments were considered statistically significant when p < 0.05.

**Results:**

A total of eight open-label RCTs were included, but only three (37.5%) of them published concomitant treatments. In two studies (ACCORD and ADVANCE), a statistically significant difference was observed between the two groups for aspirin (p = 0.02) and ACEIs (p = 0.02).

**Conclusions:**

Few concomitant treatments were published in this sample of open-label RCTs. We cannot completely eliminate an observer bias for these studies. This bias probably influenced the results to an extent that has yet to be determined.

## Background

In patients with type 2 diabetes (T2D), the efficacy of blood-glucose control is generally based on the UKPDS study [1]. The main results of this randomized study were published in 1998 and led to international guidelines on the treatment of type 2 diabetes [2]. It showed the efficacy of intensive blood-glucose control on the onset of microvascular complications. And also showed that metformin was efficacious against macrovascular complications and overall mortality in overweight patients [3]. However, even though UKPDS was randomized, this study is controversial because of its methodology and the publication of its results [4-6]. There was a risk of observer bias because the open-label study did not have any placebo group. The first potential problem lies in differences in the care management between the two groups throughout the study, combined with an imbalance in the prescription of concomitant treatments that may have influenced outcome measures [7]. This risk of bias, which is particularly high in open-label studies, can also occur in placebo-controlled, double-blind RCTs. For example in the FIELD study [8], the intake of statins is much bigger in the placebo group (36% vs 19%, p < 0.0001), which could partly explain why there is no significant difference for the primary endpoint. The consequences of such an imbalance in concomitant treatments between study groups may be particularly important since the studied outcomes are influenced by these treatments. In T2D, some antihypertensive and cholesterol-lowering drugs are effective against microvascular complications [9,10] and/or cardiovascular mortality [11,12]. Similarly, aspirin has a proven efficacy against the risk of having a coronary event in high-risk cardiovascular patients [13]. Because of this, we wondered how these concomitant treatments were reported in clinical trials on intensive blood-glucose control treatments in T2D. Our objective was also to compare concomitant treatments prescribed in each group in order to assess the possible confounding effect they may have had.

## Methods

We previously performed a systematic review using Medline, Embase, and the Cochrane Library (from January 1950 to July 2010). RCTs which were randomized, assessing the efficacy of intensive glucose lowering treatment (oral or insulin) versus a standard treatment (standard care), less intensive glycaemic lowering treatment, or placebo (intensive glycaemic treatment could be defined either by a specified HbA1c target or by treatment intensification); trials using clinically relevant outcomes; and participants aged 18 or older with type 2 diabetes were included [14]. We analyzed the articles and supplemental documents (web appendices) of RCTs included in our meta-analysis that evaluated the efficacy of intensive blood-glucose control [14]. We especially looked for the intake of ACEIs, AIIRAs, fibrates, statins, and aspirin which have a proven efficacy on diabetic complications [9-13]. When the p-value for concomitant treatments was not specified in the publications, it was directly calculated and a statistical significance of 0.05 was determined. Authors were contacted for additional data when necessary.

## Results

A total of eight open-label RCTs were found with the systematic review [1,3,15-20]. Only two publications specified concomitant treatments received by patients during the study. However, they did not publish data about all five therapeutic classes of interest (see the ACCORD and ADVANCE studies in Table 1) [18,19]. We contacted the authors of all trials, and received additional data from one study [16] (see Table 2). In total, only three studies (37,5%) reported data about concomitant treatments. In the ADVANCE study, data is only available on 86% of included patients [18]. There is a statistically significant difference (p = 0.02) in taking aspirin, which was more prescribed in the intensively treated group (Table 3). In the ACCORD study, data is available for 96% of included patients [19]. The intake of ACEIs is significantly more frequent in the conventional treatment group (p = 0.02) (Table 3). In the Kumamoto study, there was no statistically significant difference between groups (Table 3) [21].

**Table 1:** Concomitant treatments published in study reports

	**ADVANCE **[17]	**ACCORD **[18]
Antihypertensive drugs	+	+
ACEIs	-	+
ARBs	-	-
Statins	+	+
Fibrates	+	-
Aspirin	+	+

*ACEIs* angiotensin-converting enzyme inhibitors.

*ARBs* angiotensin-receptor blockers.

**Table 2:** Concomitant treatments in the Kumamoto study

	**MIT group**	**CIT group**
	**(multiple insulin injection) (N=55)**	**(Conventional insulin therapy group) (N=55)**
Statins	6	6
Fibrates	2	3
Aspirin	0	0
Antiplatelet therapy	1	2
ACEIs	7	10
ARBs	0	0

*ACEIs* angiotensin-converting enzyme inhibitors.

*ARBs* angiotensin-receptor Blockers.

**Table 3:** Differences in concomitant treatments between groups

	**Intensive (%)**	**Standard (%)**	**P value**
ADVANCE [17]			
-Antihypertensive drugs	88.9	88.4	0.44
-Statins	45.6	47.7	0.09
-Other cholesterol-lowering drugs	7	7	0.90
-Aspirin	57	54.9	**0.02**
-Other anti-aggregating drugs	7.1	6.2	0.07
ACCORD [18]			
-Anti-hypertensive drugs	91	92	0.06
-ACEIs	69.7	71.9	**0.02**
-Beta blockers	47.5	48.6	0.27
-Statins	88	87.6	0.54
-Aspirin	75.5	75.5	0.98
Kumamoto [16]	11	11	1
-Statins	3.6	5.4	0.66
-Fibrates	0	0	-
-Aspirin	1.8	3.6	0.60
- Antiplatelet therapy	12.7	18.1	0.45
-ACEI	0		-
-ARB			

*ACEI* angiotensin-converting enzyme inhibitors.

*ARB* angiotensin-receptor blockers.

## Discussion

Our study highlights the lack of publications on concomitant treatments in trials assessing intensive blood-glucose control treatments in T2D. Only three out of eight RCTs (37.5, the ACCORD, ADVANCE, and Kumamoto (after direct contact with authors) studies reported the intake of these treatments without specifying the drugs, even though their efficacy on outcome measures had been proven. In two studies (ACCORD and AVANCE), statistically significant differences at 5% were seen in both groups treated with specific medications without controlling to what extent they influence study results. This lack of data is harmful, because the interpretation of study results may be distorted and lead to incorrect recommendations for clinical practice. Pooling proportions of cointerventions and looking for an interaction between differences in cointerventions and the effect of glycemic control on outcomes is feasible using meta-analysis and meta-regression techniques. This would help us reach our second objective, "if concomitant treatments are reported in clinical trials, to assess their possible confounding effect on outcome." However, this would require a minimum of 5 trials for each covariate. We were only able to retrieve data on concomitant treatments for three trials (two published and one obtained from the authors). Therefore, we felt that meta-regression would not be appropriate and would give unrobust results [22].

The example of UKPDS 33 [1] is a prime example of this. When it was published in The Lancet in 1998, it showed that intensive blood-glucose control was effective against the onset of microvascular complications and long-term macrovascular complications. Yet, the only outcome with a statistically significant change was the “retinal photocoagulation” outcome: RR = 0.71; CI 95% [0.53-0.96]. This outcome was added during the study and let the authors conclude that the treatment was effective against all diabetic complications: “any diabetes-related endpoints” (RR = 0.88 ; CI 95% [0.79-0.99]). There was also a difference in blood pressure (BP) between some groups: at six-year follow-up, the chlorpropamide-treated group showed a mean BP that was much higher than other groups (143/82 mmHg vs 138/80 mmHg, p < 0.001). UKPDS authors emphasized that the proportion of patients treated with an antihypertensive drug was different (p = 0.022) depending on the group: 43% for the chlorpropamide-treated group compared to 34%, 36% and 38% in other groups (respectively due to lifestyle and diet guidelines, glibenclamide, and insulin). Yet, UKPDS 38 [8] showed that treating BP could help reduce the risk of developing diabetic retinopathy. The double-blind, placebo-controlled DIRECT-2 RCT also showed that candesartan increases the rate of retinopathy regression in T2D by 34% (RR = 1.34; CI 95% (1.08-1.68)) [23]. In insulin-dependent (ID) diabetes, enalapril and losartan also proved to be effective on diabetic retinopathy regardless of BP (OR = 0.35 : CI 95% [0.14-0.85], OR = 0.30 ; [0.12-0.73] respectively) [24]. The FIELD [9] and ACCORD-Lipid [25] studies (two double-blind placebo-controlled RCTs) showed that fenofibrate was effective on retinopathy in T2D, regardless of the decrease in serum lipids. The efficacy of fenofibrate on this outcome measure seemed even higher than for blood-glucose control. Since UKPDS 34 was published, metformin has been considered to be the most effective treatment for overweight patients with T2D [3]. For overall mortality, the risk ratio of metformin compared to lifestyle and diet guidelines was 0.64, CI 95% [0.45-0.91]. However, a recent meta-analysis showed that metformin was not necessarily more effective than other treatments [26]. So, the positive result observed in UKPDS 34 may just be artificial, especially since in the same study, only the combination of metformin and sulfonamides was deleterious compared to sulfonamides alone (for overall mortality: RR = 1.6, CI 95% [1.02-2.52]). It would have been essential to know which concomitant treatments were present in this study. In a letter to the authors of UKPDS after the 10-year follow-up publication, the question of concomitant treatments came up: “Information on accompanying treatment during the study is necessary in order to interpret the mortality data.” [27] Surprisingly, UKPDS authors did not respond to this [28].

A lack of blinding may overestimate the effect studied from 17% to 34% [29-31]. However, not blinding can also lead to a lack of difference because the control group does not “stay constant.” For instance, the MRFIT study observed the effect of the multifactorial care management of cardiovascular risk on 12,000 patients compared to usual care. After seven years of follow-up, no difference between patient groups was observed for overall mortality or coronary events. One of the authors’ hypotheses was that the control group (usual care) had changed its health habits. Smoking had dropped from 59% to 46%, diastolic BP from 91 to 84 mmHg, and antihypertensive drug intake had increased from 19% to 47%. Because the cardiovascular risk of this control group decreased, the study had insufficient statistical power and could not demonstrate a statistically significant difference [32]. Yudkin [33] and Gale [34] call this phenomenon the “Hawthorne effect”: the study itself may change patients’ and doctors’ behavior. This is more of a problem in open-label studies where patients and doctors know what the study drug is. So it is appropriate that CONSORT 2010 recommends in Section 11b (on the blinding of RCTs) that co-intervention similarities [35] must be described and verified, which was not required in 2001 [36]. Concerning T2D treatment, the demonstration of blood-glucose control efficacy seems to be affected by the lack of publications on concomitant treatments whose effect on diabetic complications is already proven. However, it remains to be determined to what extent the results are affected by this bias.

## Conclusions

Few concomitant treatments were published in this sample. There is a potential risk of observer bias in studies assessing the efficacy of blood-glucose control in T2D.

## Competing interests

The authors do not have any competing interests to declare.

## Authors’ contributions

RB, CC, and FG conceived the study. RB, IS, and SE extracted the data and reviewed the selected papers. RB and CC performed the statistical analysis. RB, IS, SE, CC, FG, and TBA drafted the manuscript. MC, BK, CC, TBA, and FG helped interpret the results. All authors read and approved the final manuscript.

## Authors’ information

RB: general practitioner and lecturer

IS: general practitioner and lecturer

SE: general practitioner and lecturer

MC: consultant

TBA: cardiologist, pharmacologist, and lecturer

BK: pediatric pharmacologist and professor

CC: endocrinologist, pharmacologist, and lecturer

FG: cardiologist and professor

## Pre-publication history

The pre-publication history for this paper can be accessed here:

http://www.biomedcentral.com/1471-2288/13/107/prepub
